# Parchment Paper

**Published:** 1871-01

**Authors:** 


Parchment Paper.—To convert paper into vegetable parchment, immerse it for a few seconds in a cold mixture of one volume water and two volumes sulphuric acid. Wash out rapidly by plunging in a large quantity of cold water, and finally remove all traces of the acid by further immersion in water to which a small quantity of amonia has been added. To prevent the tendency to contract when drying, it should be attached to a frame while wet, or allow it to dry under a press. Paper prepared in this way is very transparent, and can be used for copying by tracing. It is extensively employed as a substitute for parchment made from sheepskin in diplomas, certificates, patents, and for envelopes.—*Chemical News.*

